# Transpiration and Water Use Efficiency of Mediterranean *Eucalyptus* Genotypes Under Contrasting Irrigation Regimes

**DOI:** 10.3390/plants14142232

**Published:** 2025-07-19

**Authors:** Juan C. Valverde, Rafael A. Rubilar, Alex Medina, Matías Pincheira, Verónica Emhart, Yosselin Espinoza, Daniel Bozo, Otávio C. Campoe

**Affiliations:** 1 Cooperativa de Productividad Forestal, Departamento de Silvicultura, Facultad de Ciencias Forestales, Universidad de Concepción, Victoria 500, Concepción 4030555, Chile; juvalverdeo@udec.cl (J.C.V.);; 2 Forest Engineering School, Tecnológico de Costa Rica, Cartago 302101, Costa Rica; 3 Centro Nacional de Excelencia para la Industria de la Madera (CENAMAD)-ANID BASAL FB210015, Pontificia Universidad Católica de Chile, Vicuña Mackenna 4860, Santiago 7820436, Chile; 4 Forestal Mininco S.A., Avenida Alemania 751, Los Ángeles 4440000, Chile; 5 Forest Productivity Cooperative, Departamento de Ciências Florestais, Universidade Federal de Lavras, Lavras 37200, MG, Brazil

**Keywords:** productivity, water balance, sap flow, transpiration, forest management

## Abstract

Water scarcity is a key constraint for commercial *Eucalyptus* plantations, particularly given the increasing frequency of droughts driven by climate change. This study assessed annual transpiration (Tr) and water use efficiency (WUE) across eight genotypes subjected to contrasting irrigation regimes (WR). A split-plot design was implemented, comprising two irrigation levels: high (maintained above 75% of field capacity) and low (approximately 25% above the permanent wilting point). The genotypes included *Eucalyptus globulus* (EgH, EgL), *E. nitens × globulus* (EngH, EngL), *E. nitens* (En), *E. camaldulensis × globulus* (Ecg), *E. badjensis* (Eb), and *E. smithii* (Es). Between stand ages of 7 and 9 years (2020–2023), we measured current annual increment (CAI), leaf area index (LAI), Tr, and WUE. Under high WR, CAI ranged from 8 to 36 m^3^ ha^−1^ yr^−1^, Tr from 520 to 910 mm yr^−1^, and WUE from 0.7 to 2.9 kg m^−3^. Low irrigation reduced CAI by 5–25% and Tr by 10–35%, while WUE responses varied across genotypes, ranging from a 12% decrease to a 48% increase. Based on their functional responses, genotypes were grouped as follows: (i) stable performers (Es, Ecg, Eb) exhibited high WUE and consistent Tr under both WR; (ii) partially plastic genotypes (EgH, EngH) combined moderate reductions in Tr with improved WUE; and (iii) water-sensitive genotypes (EgL, EngL, En) showed substantial declines in Tr alongside variable WUE gains. These findings underscore the importance of selecting genotypes with adaptive water-use traits to improve the resilience and long-term sustainability of *Eucalyptus* plantations in Mediterranean environments.

## 1. Introduction

Water availability is a major driver of *Eucalyptus* plantation productivity [1,2], yet drought sensitivity remains a crucial constraint in regions with recurrent water scarcity [3,4]. Climate change further intensifies these limitations, increasing the frequency and severity of droughts [5,6] due to rising temperatures and altered rainfall patterns [7,8]. According to projections by the Intergovernmental Panel on Climate Change (IPCC), even moderate warming scenarios (0.5 °C to 2.0 °C by 2100) could exacerbate water scarcity and threaten the viability of reforestation programs, particularly in areas already marginal for intensive silviculture [9,10].

These emerging climate challenges underscore the need for adaptive strategies to improve the resilience of *Eucalyptus* plantations [11,12]. In this context, the identification and deployment of drought-tolerant genotypes has become a strategic priority [13,14]. While advances in genetic improvement and intensive silviculture have greatly enhanced Eucalyptus productivity and enabled its expansion into marginal regions, our understanding of how these practices influence hydrological processes (particularly under drought conditions) remains limited [15,16]. This gap is especially relevant in water-scarce areas, where forest plantations can alter groundwater recharge and increase competition with other land uses [12,17]. Therefore, identifying drought-tolerant genotypes with high water use efficiency (WUE) that could sustain growth under limited water availability is essential for ensuring water security in forested landscapes vulnerable to climate change [3,18,19].

Transpiration is a key physiological trait underlying genotypic performance under drought, as it reflects the water loss inherently associated with photosynthetic carbon gain [2,20]. In *Eucalyptus* plantations, higher transpiration rates are often associated with increased productivity [21,22]. However, under limited water availability, maintaining an optimal balance between water loss and biomass accumulation becomes critical [23,24]. In this context, water use efficiency (WUE, defined as the amount of biomass produced per unit of water transpired annually) has emerged as a key factor for evaluating drought adaptation and is increasingly incorporated into Eucalyptus genetic improvement programs [25,26]. Although WUE has been widely studied in agricultural and forestry systems, long-term evaluations in forest species remain limited [17,22].

Genotypic variation in water use strategies (e.g., differences in transpiration rates, root development, and stomatal opening and closure) plays a fundamental role in drought tolerance [27,28,29]. Additionally, silvicultural interventions such as thinning, fertilization, and optimized spacing could influence WUE by altering canopy structure and enhancing sunlight capture and photosynthesis [30,31]. However, the extent to which these genotypic traits interact with environmental constraints remains poorly understood [32]. This knowledge gap is especially critical in Mediterranean climates, where summer droughts are expected to increase in frequency and duration under future climate scenarios [11,33].

To support adaptive forest management in water-limited environments, it is crucial to evaluate how genotypes differ in their ability to balance water use and biomass production under contrasting irrigation regimes [1,34]. Integrated physiological indicators (e.g., transpiration measured via sap flow, soil water uptake, and stand-level WUE) can provide valuable insights into genotypic responses to water stress [2,35]. For example, de Bastos et al. [4] reported that *E. saligna* exhibited high annual transpiration but relatively low WUE in moist environments, while *E. dunnii* demonstrated higher water efficiency under similar conditions. These context-dependent trade-offs highlight the need for long-term, multi-genotype field evaluations to identify resilient *Eucalyptus* genotypes [16].

Therefore, this study evaluated the effects of prolonged water availability on annual transpiration and water use efficiency in eight *Eucalyptus* genotypes: *E. globulus* (EgH and EgL), *E. nitens × globulus* (EngH and EngL), *E. nitens* (En), *E. camaldulensis × globulus* (Ecg), *E. badjensis* (Eb), and *E. smithii* (Es). These genotypes were selected to represent a range of taxonomic lineages and growth performance levels previously observed under Mediterranean conditions. We hypothesized that: (i) genotypes would exhibit significant differences in transpiration and WUE in response to contrasting irrigation regimes; (ii) genotypes with higher WUE and moderate transpiration would better sustain productivity under drought; and (iii) prolonged water deficit would accentuate genotypic contrasts, facilitating the identification of resilient *Eucalyptus* genotypes. The findings aim to inform genotype selection and plantation design strategies that enhance the adaptive capacity of plantations under future climate scenarios.

## 2. Results

### 2.1. Effects of Genotype, Irrigation Regime, and Age

The genotype (Gen) had a significant effect on all measured variables (*p* < 0.01; Table 1). Age influenced all traits except LAI, indicating structural canopy stability after closure. The irrigation regime (WR) significantly affected LAI, transpiration (Tr), and water use efficiency (WUE), but not CAI or stem biomass (W_stem_), suggesting a stronger effect on functional traits than on structural traits. Significant interactions between genotype and water availability (Gen × WR) across variables reflect genotype-specific responses to water availability. Only CAI and WUE showed Age × Gen interactions, while no effects were detected for Age × WR or Age × Gen × WR. These patterns underscore consistent genotypic differences over time and a predominant influence of WR on short-term physiology rather than cumulative growth.

### 2.2. Stand Productivity

**Figure 1:**
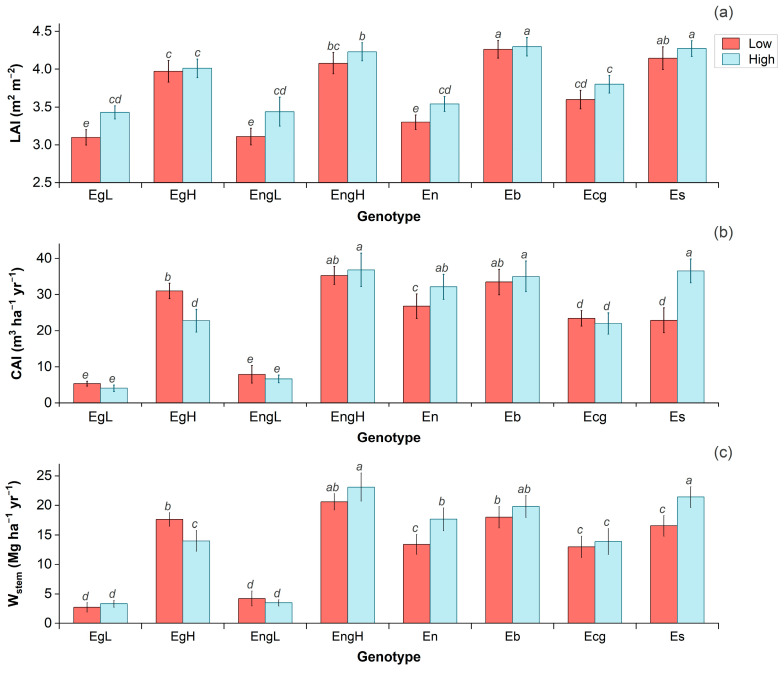
Leaf area index (LAI; (**a**)), current annual increment (CAI; (**b**)), and stem biomass increment (W_stem_; (**c**)) of eight *Eucalyptus* genotypes evaluated between ages 7 and 9 under contrasting irrigation regimes (low and high). (Error bars above each bar mean represents its standard error. Different letters denote significant differences among genotype × irrigation regime interactions at *p* < 0.05.)

Leaf area index (LAI) responses to the WR varied notably among genotypes (Figure 1a). EgH, EngH, Ecg, Eb, and Es did not exhibit significant differences between WR treatments (*p* > 0.05), indicating stable canopy development under water-limited conditions. Among these, EngH, Eb, and Es reached the highest LAI values (>4.0 m^2^ m^−2^), while Ecg and EgH showed intermediate levels (~3.7 m^2^ m^−2^). In contrast, EgL, EngL, and En experienced significant LAI reductions (15–22%) under low WR, with mean values declining to approximately 3.35 m^2^ m^−2^.

The current annual increment (CAI) and stem biomass increment (W_stem_) varied among genotypes (Figure 1b,c). EgH was the only genotype that increased CAI and W_stem_ under low WR, with a gain of ~18%. In contrast, En and Es exhibited reductions of 9–20% in CAI and 12–25% in W_stem_ under water stress. The remaining genotypes (EgL, EngL, EngH, Ecg, and Eb) maintained stable across WR and were functionally grouped by productivity: low (EgL, EngL; <10 m^3^ ha^−1^ yr^−1^ and <10 Mg ha^−1^ yr^−1^), moderate (Ecg; ~23 m^3^ ha^−1^ yr^−1^ and ~15 Mg ha^−1^ yr^−1^), and high (EngH, Eb; >30 m^3^ ha^−1^ yr^−1^ and >18 Mg ha^−1^ yr^−1^).

### 2.3. Transpiration and Water Use Efficiently

**Figure 2:**
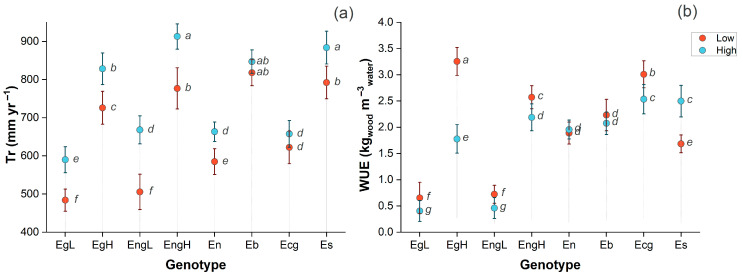
Annual transpiration (Tr; (**a**)) and water use efficiency (WUE; (**b**)) of eight *Eucalyptus* genotypes evaluated between ages 7 and 9 under contrasting irrigation regimes (low and high). (Error lines represent the standard error of the mean; different letters indicate significant differences between genotype × irrigation regime interactions at *p* < 0.05).

Tr (Figure 2a) showed significant Gen × WR interactions (*p* < 0.05). Among the evaluated genotypes, EgH and EngH showed the largest reductions in Tr under low WR, with decreases of approximately 150–200 mm yr^−1^ (approximately 20–25% lower than under high WR). EgL, EngL, and En exhibited intermediate reductions of around 100–150 mm yr^−1^ (15–20%). In contrast, Es and Ecg maintained comparable Tr across treatments, with values consistently near 800–900 mm yr^−1^, suggesting greater stability of water flux under varying irrigation conditions.

WUE also showed significant Gen × WR interactions (*p* < 0.05) (Figure 2b). Under low WR, EgL and EngL exhibited the largest increases in WUE, rising from below 1.0 kg m^−3^ to approximately 1.5–2.0 kg m^−3^, reflecting an adaptive shift toward greater water use efficiency under reduced water supply. Es maintained a high WUE across both treatments, around 3.0–3.5 kg m^−3^, indicating stable and efficient water use. Ecg and Eb also showed minimal variation between irrigation regimes, with WUE values of 2.5–3.0 kg m^−3^, suggesting functional stability. In contrast, EgH and En presented only moderate increases in WUE under low WR, reaching approximately 2.0 kg m^−3^, highlighting a less pronounced adjustment in efficiency compared to other genotypes.

Overall, based on the combined responses of Tr and WUE, three functional groups could be distinguished: (i) stable genotypes (Es, Ecg, and Eb), which maintained consistent Tr and high WUE across both irrigation regimes, indicating strong functional stability; (ii) partially plastic genotypes (EgH and EngH), which showed substantial reductions in Tr together with moderate increases in WUE, reflecting an intermediate capacity to adjust to water limitation; and (iii) water-susceptible genotypes (EgL, EngL, and En), characterized by intermediate reductions in Tr along with pronounced increases in WUE, suggesting an active water-saving strategy to sustain growth under low-WR conditions.

### 2.4. Stability in Transpiration and Water Use Efficiency

**Figure 3:**
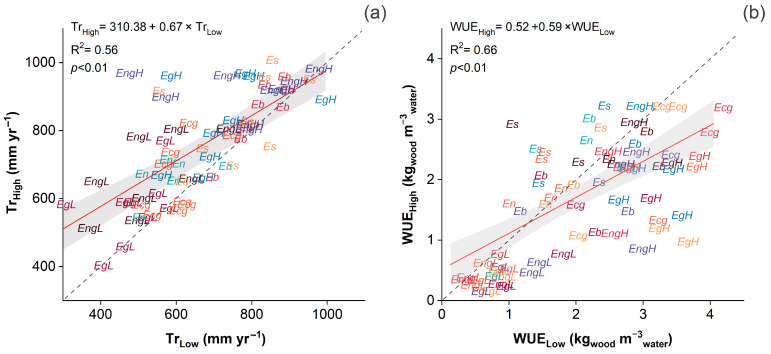
Stability of annual transpiration (Tr; (**a**)) and water use efficiency (WUE; (**b**)) of eight *Eucalyptus* genotypes under contrasting irrigation regimes (low and high). (The segmented line represents a 1:1 relationship, the red line indicates the regression fit, and the shaded gray area denotes the 95% confidence band).

Figure 3 presents the relationship between Tr and WUE across contrasting WR. Tr (Figure 3a) showed a positive and significant correlation between high and low WR (R^2^ = 0.56, *p* < 0.01), suggesting that genotypes with higher transpiration under restricted conditions generally maintained higher fluxes under full irrigation. However, the slope below unity (0.67) indicates a proportional reduction in Tr under limited water supply. Similarly, WUE (Figure 3b) presented a significant positive association between WR (R^2^ = 0.66, *p* < 0.01), with a slope of 0.59, reflecting moderate stability in WUE but also indicating genotype-dependent shifts in water-use performance under drought.

### 2.5. Relationship Between Transpiration and Productivity

**Figure 4:**
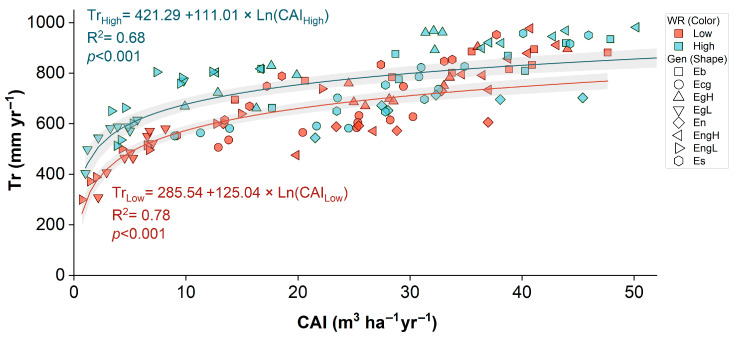
Relationship between annual transpiration (Tr) and current annual increment (CAI) in eight *Eucalyptus* genotypes under contrasting irrigation regimes (low and high). (Shaded areas indicate 95% confidence intervals for the fitted models).

The relationship between Tr and CAI (Figure 4) showed a significant positive logarithmic association under both irrigation regimes, with a stronger fit under low WR (R^2^ = 0.78, *p* < 0.001) than under high WR (R^2^ = 0.68, *p* < 0.001). Despite the similar functional form, the steeper slope observed under low WR indicates a higher marginal transpiration cost per unit of biomass produced, reflecting reduced WUE under drought. This difference in slope parameters suggests distinct physiological adjustments to water availability. Genotypic dispersion along the curve further highlights contrasting water-use strategies, with some genotypes maintaining high productivity at moderate transpiration rates, while others exhibit disproportionately high water loss relative to growth. These patterns underscore the relevance of genotype selection to sustain productivity under limited water supply.

## 3. Discussion

### 3.1. Genotypic Responses to Contrasting Irrigation Regimes

Water availability is a key driver of functional differentiation among *Eucalyptus* genotypes. Even under structurally similar stands, contrasting irrigation regimes revealed clear variation in water-use traits, reflecting genotypic differences in stomatal behavior [36], rooting depth [25], and xylem properties [37,38]. These physiological strategies influence both transpiration and water use efficiency, especially under variable moisture conditions. Understanding such responses is crucial for selecting genotypes that are better adapted to projected increases in drought and climate variability [39,40].

Stable genotypes (Es, Ecg, and Eb) demonstrated consistent Tr and high WUE across contrasting WR. This stability likely reflects conservative water-use strategies, where stomatal regulation supports carbon assimilation per unit of water rather than maximizing absolute transpiration [23]. Under closed-canopy conditions, such regulation may buffer short-term fluctuations in soil moisture and help maintain internal water balance [6,41]. This functional stability may be supported by traits such as deeper or more adaptable root systems, greater stomatal sensitivity to water stress, and favorable leaf-to-sapwood area ratios [32,42], which enable sustained growth under moderate drought conditions.

Partially plastic genotypes (EgH, EngH) reduced Tr under drought while moderately improving WUE, indicating flexible water-use strategies. EgH even increased CAI under low WR, whereas EngH maintained stable growth. These patterns suggest a balanced trade-off between water savings and productivity [28,43]. Previously, Christina et al. [34] and Hakamada et al. [24] suggested that variations in xylem vulnerability, root distribution, and stomatal behavior may serve as the foundation for the plasticity observed in *Eucalyptus* genotypes, thereby enhancing their resilience in environments characterized by variable water availability.

Finally, susceptible genotypes (EgL, EngL, En) showed reduced Tr and notable WUE gains under drought, reflecting a shift toward water conservation. This strategy, possibly driven by stricter stomatal regulation or enhanced photosynthetic efficiency [2,35], may constrain maximum productivity under optimal conditions but improve resilience under stress [25,37].

### 3.2. Correlation Between Water Use and Productivity

The identified positive nonlinear association between Tr and CAI across different WR shows that stand-level productivity in mature *Eucalyptus* plantations is intrinsically linked to water fluxes. Notably, the more pronounced slope observed under low WR suggests a greater marginal growth response per unit of transpired water, a phenomenon frequently documented in semiarid and Mediterranean systems where vegetation operates near hydraulic thresholds [10,38]. In such conditions, even small increases in water availability may substantially enhance carbon assimilation and growth efficiency [11,32].

The observed variability in the Tr–CAI relationship among different genotypes indicates that water usage alone does not entirely account for the differences in productivity. In addition to water flux, elements such as phenological plasticity, hydraulic conductance, and nutrient uptake efficiency are likely significant contributors to these genotype-specific outcomes [44,45,46]. Furthermore, variation in allometric traits, including sapwood-to-leaf area ratios and fine-root turnover, may affect the integration between water transport and growth [6,32]. These findings underscore the importance of a comprehensive evaluation of hydraulic, structural, and metabolic traits to predict and enhance genotype performance under varying water conditions [47,48].

### 3.3. Implications for Forest Management

With droughts becoming more frequent and severe in many Eucalyptus plantation areas [10], selecting genotypes that maintain productivity while using water efficiently is crucial to reduce the risk of stand mortality and yield decline [44]. The functional classification proposed here provides a framework to align genotype choice with site-specific water constraints, supporting climate-resilient plantations while minimizing adverse impacts on groundwater recharge, streamflow, and ecological interactions [2,3,49]. Such genotype–environment matching is essential for sustainable forest management under climate change [23,24].

Incorporating water-use traits, hydraulic safety margins, and plasticity indices into breeding programs will be increasingly important [8,18]. Selection efforts focused solely on growth or wood traits may neglect key adaptive attributes required in future climates [6,25]. Including traits such as stomatal control, rooting depth, and xylem resistance in selection criteria could improve plantation performance under water-limited conditions [4,34]. These considerations should be complemented by silvicultural practices (e.g., soil conservation, weed management, thinning, and pruning) that optimize resource use and reduce competition for water [27,44].

Broader management strategies should also support genotype performance [44]. Enhancing soil organic matter and applying mulch could improve moisture retention, while timely weed control limits water loss to competing vegetation [20,28]. Thinning and pruning could help balance canopy development with soil water availability, maintaining hydraulic safety margins [46,47]. Finally, deploying functionally diverse genotype mixtures in mosaic or mixed plantations may improve system stability, buffer climate-related risks, and contribute to adaptive, water-efficient forestry [16,32].

## 4. Materials and Methods

### 4.1. Study Site

**Figure 5:**
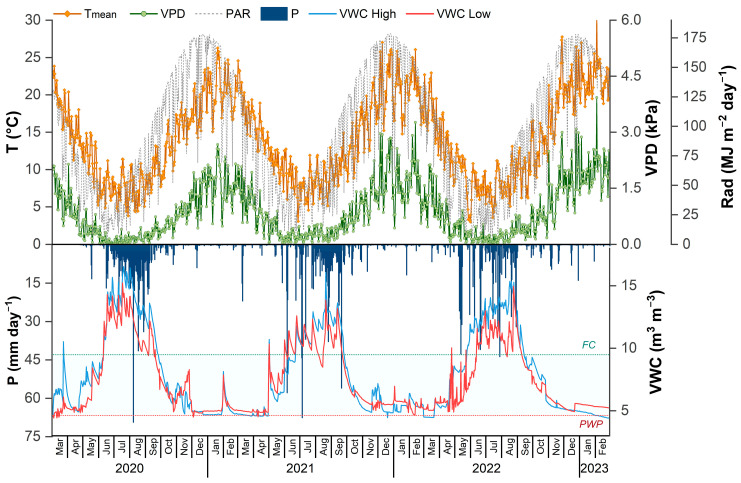
Temperature (T), vapor pressure deficit (VPD), solar radiation (Rad), precipitation (P), and volumetric water content (VWC) evaluated between 2020 and 2023 (7 and 9 years old).

**Figure 6:**
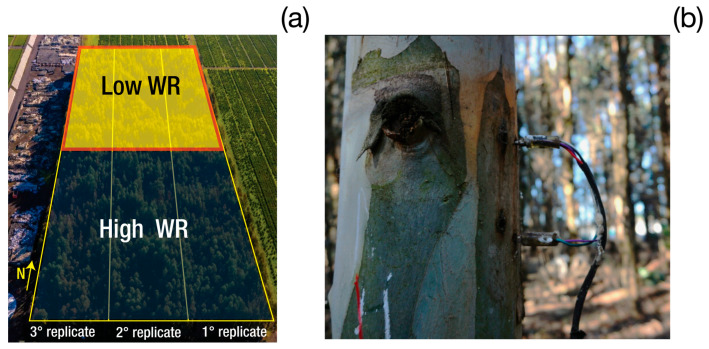
Aerial image of the experimental plantation showing the distribution of tree replicates under high and low irrigation regimes (**a**). Installation of a Granier-type sap flow sensor on a *Eucalyptus* stem without the standard thermal insulation (polystyrene and aluminum foil) (**b**).

The study was conducted in Yumbel, Biobío Region, Chile (37°08′00.01″ S, 72°27′34.70″ W), at an average elevation of 124 m above sea level. The local climate is classified as warm-summer Mediterranean (*Csb*) according to the Köppen–Geiger system [50]. The site presents a mean annual temperature of 13.6 °C, with monthly averages ranging from 6.1 °C in July to 22.8 °C in January (Figure 5). The mean annual precipitation is 1262 mm, concentrated primarily between March and September, corresponding to the austral autumn and winter seasons. Annual solar radiation varies from 5.5 to 32.1 MJ m^−2^ day^−1^, and relative humidity ranges between 20% and 60% during the growing season (Figure 6). Soil water content (SWC) and meteorological variables were monitored continuously using TDR CS650 sensors (Campbell Scientific, Logan, UT, USA), and a Vantage Pro2 (Davis Instruments, Hayward, CA, USA) weather station was installed on site.

The soil is classified as Dystric Xeropsamments [51], characterized by a sandy texture, high leaching potential, and flat topography. Within the top meter of the soil profile, texture analysis showed sand contents ranging from 86.6% to 94.3%, silt from 3.53% to 12.3%, and clay from 0.9% to 2.06%, with an average bulk density of 1.44 g cm^−3^ (details in Rubilar et al. [48]). The permanent wilting point and field capacity in this layer were estimated at 5.4% and 9.8%, respectively (Figure 5).

### 4.2. Experimental Design

The experiment was established in August 2013 using a split-plot design with three blocks (replicates), where the irrigation regime (WR) was assigned to the main plots and genotype (Gen) to the subplots (Figure 6a). Prior to planting, the soil was subsoiled to a depth of 80 cm to improve rooting conditions (Rubilar et al. [48]). Two WR levels were evaluated: high (soil water content > 75% of field capacity) and low (~25% of field capacity). Drip irrigation was applied from November to March (spring to summer), with annual adjustments made based on precipitation and evapotranspiration.

Each block included two main plots (high WR and low WR), which were distributed across two adjacent sectors of the experimental area (Figure 6a), based on irrigation system layout and the homogeneity of the site. This configuration ensured effective implementation of the irrigation treatments while maintaining environmental uniformity and facilitating operational control.

Regarding the Gen factor, the original trial included 30 *Eucalyptus* genotypes from the CMPC and ARAUCO breeding programs. Clonal plants, six months old at establishment, were planted at a spacing of 3 × 2 m (1667 trees ha^−1^). Each experimental unit consisted of 25 trees arranged in a 5 × 5 grid, with a central subplot of 9 trees (3 × 3) used for measurements to minimize edge effects. This experiment is part of the EuCarbHydro project, which aims to assess long-term genotypic responses to irrigation regimes in terms of growth, water use, and carbon allocation. For the present analysis, eight genotypes were selected to represent a productivity gradient: *E. globulus* (EgL: low-yield, EgH: high-yield), *E. nitens × globulus* (EngL: low-yield, EngH: high-yield), *E. nitens* (En), *E. badjensis* (Eb), *E. camaldulensis × globulus* (Ecg), and *E. smithii* (Es).

### 4.3. Stand Growth

The study focused on the mature stage of the plantation, after canopy closure, during a three-year period corresponding to stand ages 7, 8, and 9 (March 2020–February 2023). Diameter at breast height (DBH) and total height (TH) were measured seasonally in each internal plot (*n* = 27 trees per WR × Gen). Tree volume was estimated using the Schumacher and Hall (1933) model, previously parameterized for these genotypes (Valverde et al. [47]). Seasonal growth was calculated as the difference between consecutive measurements, and the current annual increment (CAI) was calculated as the difference between the start and end of each growth year.

Leaf area index (LAI) was measured monthly using an LAI-2200C canopy analyzer (LI-COR Biosciences, Lincoln, NE, USA) configured with two optical wands and a console equipped with a 10° field cap to reduce stem interference. Measurements were taken between 08:00 and 10:00 (UTC −3:00) under uniform overcast conditions. Six readings per internal plot were collected, both within and between rows (*n* = 18 measurements per WR × Gen × month).

### 4.4. Transpiration and Water Use Efficiency

Sap flow was estimated using the heat dissipation method, as described by Granier [52,53] (Figure 6b). Six trees were selected for each genotype × WR combination to represent the distribution of DBH accurately. The sensors, custom-built with a diameter of 20 mm in accordance with the Hubbard et al. [33] design, were installed at approximately ~1.4 m height, insulated with polystyrene and aluminum wrapping, and connected to CR1000 dataloggers equipped with AM16/32 multiplexers (Campbell Scientific, Inc.). Each set-up consisted of a heated and a reference thermocouple, positioned with a vertical spacing of 10–15 cm. Temperature differences (ΔT) were recorded every 15 min, and the sap flux density (*J*, cm^3^ cm^−2^ s^−1^) was determined using Equation (1).

(1) $$J=11899\times 10^{-6}\times {\left(\frac{∆T_{max}-∆T}{∆T}\right)}^{1.231}$$

where *J* is the sap flux density in cm^3^ cm^−2^ s^−1^, ΔT_max_ is the maximum temperature difference observed during periods of 10 days, and ΔT is the specific differential measured.

As in other long-term sap flow studies, occasional data gaps were observed in the time series due to sensor malfunction, datalogger interruptions, or environmental interferences [54,55]. To maintain continuity and avoid data loss bias, we implemented a trigonometric regression approach adapted for cyclical environmental datasets, which has been shown to perform well in capturing the diurnal dynamics of transpiration [56,57]. This method models the sap flux signal using sine and cosine terms to reflect its periodic structure, while also incorporating key climatic predictors, such as photosynthetically active radiation (PAR) and vapor pressure deficit (VPD), which are known to strongly influence transpiration rates in *Eucalyptus* species [30,58].

Missing hourly values were reconstructed by fitting the model to complete portions of the data and then predicting values over the identified gaps. The final model explained over 80% of the variance in observed sap flux density (R^2^ > 0.80), providing robust and physiologically consistent estimates for the missing periods. Compared to linear interpolation or regressions relying solely on meteorological data, this approach improves reliability by explicitly accounting for temporal autocorrelation and preserving the diel structure of sap flow patterns [59,60].

Then, the sap flux density was scaled to the tree level, considering the sapwood area, which was estimated using genotype-specific allometric equations (Table 2). The stand-level transpiration (Tr, in mm d^−1^) was then obtained by summing the sap flux contributions of all the trees sampled and normalizing by plot area (Equation (2)). Additionally, water use efficiency (WUE) was calculated as the ratio of annual transpiration and current annual increase (CAI, kg stem biomass per hectare per year).

(2) Tr=J×SA

where Tr is transpiration in mm, *J* is the density of the sap flux in cm^3^ cm^−2^ s^−1^, and SA is the sap wood area in cm^2^ tree^−1^.

### 4.5. Statistical Analysis

Differences in growth variables, transpiration Tr, and WUE were evaluated using generalized linear mixed models (GLMMs), implemented via restricted maximum likelihood (REML). The model structure accounted for repeated measurements over three consecutive years (stand ages 7, 8, and 9) and was defined in Equation (3).

(3) $$\begin{array}{c} Y_{ijkl}=\mu +{Gen}_{i}+{WR}_{j}+A_{k}+{\left(Gen\times WR\right)}_{ij}+{\left(Gen\times Age\right)}_{ik}+{\left(WR\times Age\right)}_{jk}\\ +{\left(Gen\times WR\times Age\right)}_{ijk}+{BK}_{l}+\varepsilon_{ijkl} \end{array}$$

where Y_ijkl_ is the observed value of the response variable for the *i*-th genotype, *j*-th irrigation regime, *k*-th age, and *l*-th block; μ is the overall intercept; Gen*_i_*, WR_j_, and Age_k_ are the fixed effects of genotype, irrigation regime, and age, respectively; the terms (Gen × WR)_ij_, (Gen × Age)_ik_, (WR × Age)_jk_, and (Gen × WR × Age)_ijk_ represent the fixed-effect interactions; B_kl_∼N(0,σ^2^B_kl_) denotes the random effect of block; and ε_ijkl_∼N(0,σ^2^_ijkl_) is the residual error.

Each response variable was analyzed separately using linear models that included irrigation regime (WR), genotype (Gen), stand age (Age), and their interactions as fixed effects. Although age was incorporated into the model, it was considered relevant for interpretation only when involved in significant interactions with both WR and Gen, or in the three-way interaction (Age × Gen × WR). As none of these interactions reached statistical significance, subsequent analyses focused exclusively on the Gen × WR interaction. When this interaction was significant (*p* < 0.05), pairwise comparisons among genotypes within each irrigation regime were conducted using the Tukey–Kramer procedure to adjust for multiple testing.

To explore the functional relationships between productivity (CAI) and Tr, a nonlinear regression model was fitted (Equation (4)) based on residual mean standard error (RMSE).

(4) CAI=α+β×ln(Tr)+ε

where CAI is the current annual increment (m^3^ ha^−1^ yr^−1^); Tr is the transpiration (mm yr^−1^); α and β are the model coefficients; and ε∼N(0,σ^2^) is the residual term.

All statistical procedures were carried out in R version 4.3.3 [61], with a significance threshold set at *p* = 0.05.

## 5. Conclusions

This study demonstrates that water availability is a major determinant of physiological performance and growth in mature *Eucalyptus* genotypes under Mediterranean conditions. Significant genotypic differences in transpiration and WUE were observed across irrigation regimes, with drought accentuating these contrasts. Genotypes such as EgH and EngH maintained productivity through moderate water use and improved efficiency, highlighting their adaptive potential.

By integrating multi-year field measurements under operational conditions, this work offers a practical framework for classifying genotypes based on functional water-use responses. These findings have direct applications in selecting drought-resilient genotypes for afforestation programs, guiding irrigation planning, and designing climate-adaptive plantations in water-limited regions.

## Figures and Tables

**Table 1:** Results of the analysis of variance (*p*-values) for the fixed effects of genotype (Gen), irrigation regime (WR), age and interactions in productivity (CAI, W_stem_), leaf area index (LAI), transpiration (Tr), and water use efficiency (WUE) of eight *Eucalyptus* genotypes under contrasting irrigation regimes (low and high).

Variable	Factor						
	Age	Gen	WR	Age × Gen	Age × WR	Gen × WR	Age × Gen × WR
LAI	*ns*	˂0.001	0.03	*ns*	*ns*	0.004	*ns*
CAI	˂0.001	˂0.001	*ns*	0.01	*ns*	0.007	*ns*
W_stem_	˂0.001	0.004	*ns*	*ns*	*ns*	0.005	*ns*
Tr	˂0.001	˂0.001	˂0.001	*ns*	*ns*	0.01	*ns*
WUE	˂0.001	˂0.001	˂0.001	0.02	*ns*	˂0.001	*ns*

Note: *ns* = not significant at *p* > 0.05.

**Table 2:** Fitted sapwood equations for the eight *Eucalyptus* genotypes evaluated under contrasting irrigation regimes (low and high).

Genotype	Code	Coefficients				*p*-Value	R^2^	RMSE	AIC	BIC
		β0	SE	β1	SE					
*E. globulus* low-yield	EgL	7.15 × 10^−5^	7.39 × 10^−6^	2.01	0.04	0.001	0.99	0.019	10.01	10.00
*E. globulus* high-yield	EgH	5.71 × 10^−5^	5.89 × 10^−6^	2.08	0.03	0.001	0.98	0.018	9.99	8.89
*E. nitens × globulus* low-yield	EngL	1.46 × 10^−5^	2.28 × 10^−6^	1.91	0.05	0.001	0.98	0.025	11.12	10.55
*E. nitens × globulus* high-yield	EngH	6.87 × 10^−5^	5.28 × 10^−6^	2.02	0.02	0.002	0.97	0.033	12.22	13.01
*E. nitens*	En	7.68 × 10^−5^	1.15 × 10^−6^	1.98	0.05	0.001	0.99	0.022	10.22	10.51
*E. badjensis*	Eb	8.68 × 10^−5^	4.61 × 10^−6^	1.94	0.01	0.001	0.98	0.019	12.00	12.15
*E. camaldulensis × globulus*	Ecg	8.77 × 10^−5^	9.15 × 10^−6^	1.93	0.03	0.001	0.97	0.013	13.33	12.56
*E. smithii*	Es	5.73 × 10^−5^	2.07 × 10^−6^	2.08	0.03	0.001	0.99	0.011	10.01	9.88

## Data Availability

Data is contained within the article.
